# Construction and Characterization of the Novel Engineered Endolysin ALC005: A New Alternative Antimicrobial Against *Riemerella anatipestifer*

**DOI:** 10.3390/microorganisms14081622

**Published:** 2026-07-24

**Authors:** Hongmei Chen, Nansong Jiang, Weiwei Wang, Qizhang Liang, Qiuling Fu, Rongchang Liu, Chunhe Wan, Yu Huang, Guanghua Fu, Longfei Cheng

**Affiliations:** 1 Research Center for Poultry Diseases, Institute of Animal Husbandry and Veterinary Medicine, Fujian Academy of Agricultural Sciences, Fuzhou 350013, China; chenhongmei2025@126.com (H.C.); nansongjiang@126.com (N.J.); wangweiweihn@163.com (W.W.); 11817043@zju.edu.cn (Q.L.); qiulingfu0822@163.com (Q.F.); liurongc@foxmail.com (R.L.); chunhewan@126.com (C.W.); huangyu_815@163.com (Y.H.); 2 Fujian Key Laboratory for Prevention and Control of Avian Diseases, Fuzhou 350013, China; 3 Fujian Industry Technology Innovation Research Academy of Livestock and Poultry Diseases Prevention & Control, Fuzhou 350013, China

**Keywords:** *Riemerella anatipestifer*, cell-penetrating peptide, endolysin, receptor-binding protein, polycationic nonapeptide

## Abstract

The increasing serotypic diversity and multidrug resistance of *Riemerella anatipestifer* pose a serious threat to the poultry industry, necessitating novel antimicrobial agents. Although phage-derived endolysins offer rapid and specific bactericidal activity with low resistance potential, their efficacy against this Gram-negative pathogen is severely constrained by the outer membrane barrier, driving the need for strategies to enhance endolysin penetration. In this study, the native endolysin NA of phages vB_RanS_CRP2 was verified to lack antibacterial activity. To overcome this barrier, we constructed three chimeric proteins—ALC001, ALC005, and ALC007—by fusing a receptor-binding protein, a cell-penetrating peptide, or a polycationic nonapeptide, respectively. All three chimeras exhibited dose-dependent antibacterial activity, with ALC005 demonstrating the best performance. ALC005 achieved a lytic rate of 72.2% against the tested *Riemerella anatipestifer* strains, remained stable at 0–40 °C and pH 6–9, and was shown by transmission electron microscopy to exert its bactericidal effect by disrupting the bacterial cell envelope and inducing cell lysis. Collectively, cell-penetrating peptide fusion is a reliable and effective strategy to potentiate endolysin activity against *Riemerella anatipestifer*.

## 1. Introduction

*Riemerella anatipestifer* (*R. anatipestifer*) serves as the main causative agent of infectious pericarditis in domesticated birds such as ducks, chickens, and geese [1,2,3]. *R. anatipestifer*-associated infections bring about high sickness and death rates, creating significant economic hardship for the worldwide poultry industry [4]. For decades, antibiotic administration has been the primary approach to combat *R. anatipestifer* infections [5]. However, the emergence of at least 21 serotypes with limited cross-protection, together with the irrational use of antibiotics in production settings, has led to a sharp rise in multidrug resistance among *R. anatipestifer* strains [6]. Many antibiotics that were once effective have now failed to produce satisfactory therapeutic effects [7,8]. Thus, the urgent development of next-generation antibacterial agents against *R. anatipestifer* that are both potent and resistant to resistance evolution is imperative [9]. Their mechanism of action involves specific hydrolysis of peptidoglycan within the bacterial cell wall, which disrupts cell wall integrity and results in bacterial lysis and death [10,11].

Compared with traditional antibiotics, endolysins have significant advantages such as rapid action and high specificity, and are difficult for pathogens to develop resistance to [12]. They have been successfully applied in the prevention and treatment of infections caused by Gram-positive bacteria such as *Staphylococcus aureus* [13,14]. However, Gram-negative bacteria have a natural permeability barrier composed of the outer membrane, which can effectively prevent the contact between the endolysins and the peptidoglycan substrates, resulting in the lack of direct antibacterial activity of natural endolysins [15,16,17]. For example, a recently reported lysogenic enzyme, LysGDF21 from *R. anatipestifer*, exhibits no direct bactericidal activity when used alone and requires combination with outer membrane permeation agents to function [18]. This highlights the necessity of breaking through the outer membrane barrier for the development of therapeutic endolysins against *R. anatipestifer*.

To overcome this obstacle, researchers have developed various strategies to enhance the penetration ability of the endolysins into the outer membrane of Gram-negative bacteria. The main methods include: using outer membrane penetration agents (such as EDTA, citric acid) in combination [19,20]; fusing the endolysins with phage receptor-binding proteins (RBPs) or cell-penetrating peptides (CPPs) through genetic engineering [21,22,23]; and introducing hydrophobic groups or positively charged residues [21,24,25]. Among these, the engineering modification strategy of fusing functional elements is considered to have a more specific application prospect because it can endow the endolysins with autonomous penetration ability and avoid the use of potentially toxic chemical penetration agents. For example, fusing the endolysins with RBPs from phages can utilize their ability to specifically recognize the surface receptors of the target bacteria, mediating the precise localization and function of the endolysin [23]. Fusing CPPs or PCNPs in positive charges may non-specifically disrupt the stability of the outer membrane through electrostatic interactions, creating conditions for the endolysins to come into contact with the peptidoglycan [26,27].

Although these strategies have achieved certain success in the engineering modification of endolysins for various Gram-negative bacteria such as *Escherichia coli* and *Pseudomonas aeruginosa* [28,29], research on endolysins targeting *R. anatipestifer* remains limited [18]. There is a lack of systematic comparison of the functional element fusion modification strategies for *R. anatipestifer* endolysin, and the applicability and efficacy of different strategies on the specific strain of *R. anatipestifer* and their synergistic effects remain unclear. Based on this, this study first cloned and expressed the endolysin gene isolated and identified from phage vB_RanS_CRP2 (CRP2) of *R. anatipestifer*, and carried out engineering modification of the obtained endolysin. The antibacterial effects of three engineering strategies, namely fusion of the RBP, CPP, and polycationic nonapeptide (PCNP), were systematically compared. The aim was to screen out the optimal functional element that enhances the antibacterial activity of the endolysin against *R. anatipestifer* and to thoroughly evaluate the in vitro biological characteristics of the selected engineered endolysin, including its antibacterial spectrum, stability, and mechanism of action. This study provides a theoretical and experimental foundation for the development of novel biological agents against *R. anatipestifer*.

## 2. Materials and Methods

### 2.1. Bacterial Strains and Culture Conditions

Nineteen *R. anatipestifer* strains, five *E. coli* strains and five *Salmonella* strains were used to evaluate the antibacterial activity (Table 1). The *R. anatipestifer* phage CRP2 (GenBank accession number: OQ067112) was a laboratory-conserved phage, and its host strain was *R. anatipestifer* RAf1. *E. coli* TOP10 and *E. coli* BL21 (DE3) were used for molecular cloning and recombinant protein expression.

*R. anatipestifer* was cultured on Tryptic Soy Agar (TSA; BD Difco^TM^, Franklin Lakes, NJ, USA) plates at 37 °C for 16–18 h. Several individual colonies were then selected and inoculated into Tryptic Soy Broth (TSB;BD Difco^TM^, Franklin Lakes, NJ, USA), followed by incubation at 37 °C with shaking at 200 rpm until the OD_525_ reached approximately 0.8–1.0. *E. coli* and *Salmonella* strains were streaked onto Lysogeny Broth (LB; BD DifcoTM, Franklin Lakes, NJ, USA) agar plates and incubated at 37 °C for 16–18 h. Selected colonies were then transferred into LB and grown under the same conditions (37 °C, 200 rpm) until the OD_600_ reached approximately 0.8–1.0. Kanamycin was added to the medium at a final concentration of 50 µg/mL when necessary. All bacterial strains were stored at −80 °C for preservation.

### 2.2. Design of the Endolysin Gene Modification

**Figure 1:**
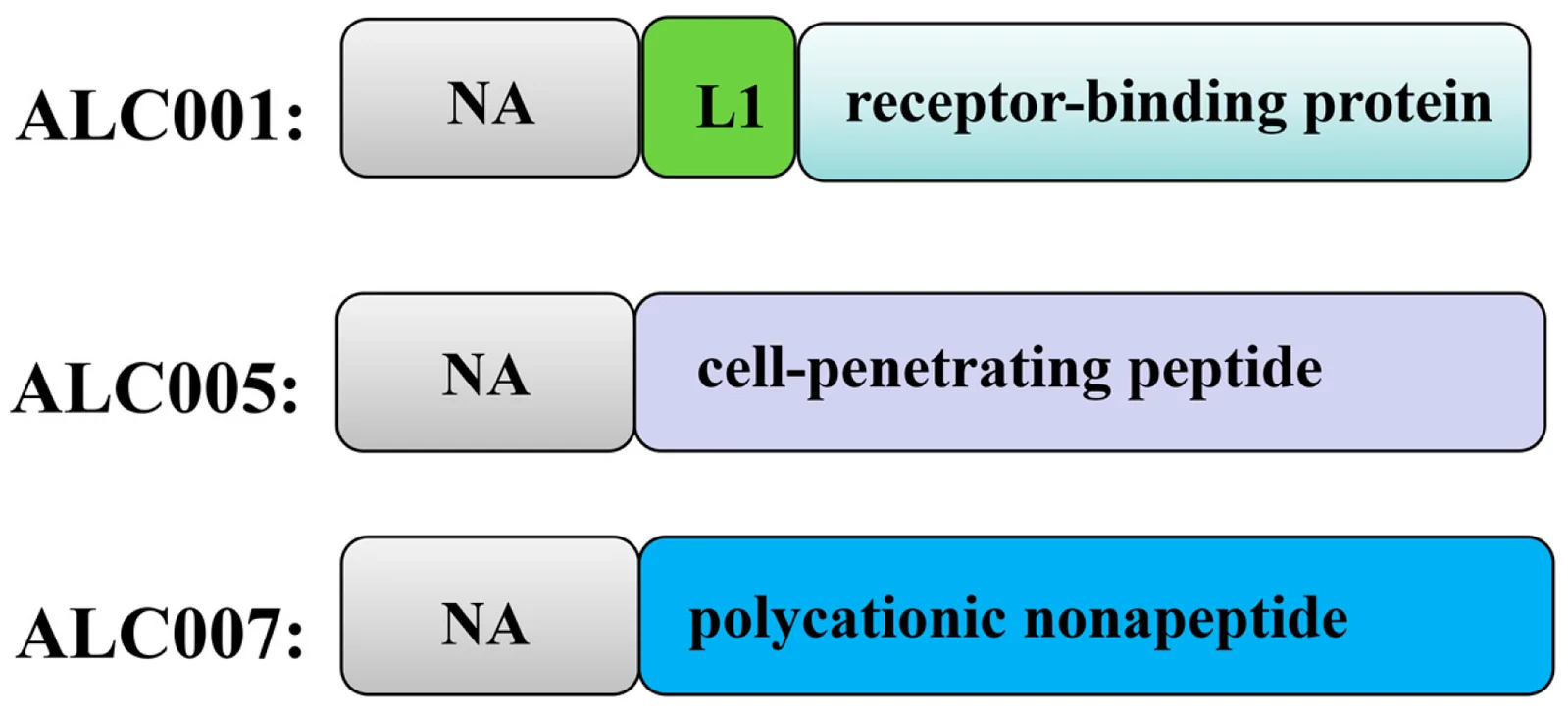
Visual representation of construction of recombinant proteins.

Four synthetic genes were constructed for protein expression (Figure 1). The native endolysin gene (designated *NA*) was obtained from the genome of *R. anatipestifer* phage CRP2. The *ALC001* gene was constructed by fusing the endolysin *NA* gene with the *RBP* gene of phage CRP2 [30] via the flexible linker encoding the amino acid sequence “AGAGAG”. The *ALC005* gene was constructed by fusing the *NA* gene with the *CPP* gene encoding the amino acid sequence “RIMRILRILKLAR”. The *ALC007* gene was constructed by fusing the *NA* gene with the *PCNP* gene encoding the amino acid sequence “KRKKRKKRK”. All synthetic genes were synthesized by Wuhan Fanpu Biotechnology Co., Ltd. (Wuhan, China)., cloned into the pET-28a expression vector using NcoI and XhoI restriction sites, with the resulting constructs containing a C-terminal 6×His tag, and subsequently transformed into electrocompetent *E. coli* BL21 (DE3) cells by electroporation.

### 2.3. Expression and Purification of Recombinant Proteins

Transformed cells were cultured in LB medium supplemented with 50 μg/mL kanamycin at 37 °C until the OD_600_ reached 0.3–0.4. Protein expression was then induced by adding 0.5 mM isopropyl-β-D-thiogalactopyranoside (IPTG; Thermo Scientific, Waltham, MA, USA), followed by incubation at 15 °C for 28 h.

After induction, the culture was centrifuged at 8000× *g* for 10 min, and the supernatant was discarded. The cell pellet was resuspended in 40 mL of lysis buffer, frozen, and subsequently subjected to sonication for 1 h. The lysate was centrifuged at 10,000× *g* for 15 min at 4 °C, and the supernatant and precipitate were collected separately. Samples were analyzed by SDS-PAGE. The polyacrylamide gel band corresponding to the target protein was excised and sent to Sangon Biotech Co., Ltd. (Shanghai, China). for mass spectrometry identification. A Ni-agarose 6FF column was packed, rinsed with 5 column volumes of deionized water, and equilibrated with 5 volumes of lysis buffer before loading the lysate supernatant. Impurities were removed by washing with 10 volumes of wash buffer, and the target protein was eluted with 5 volumes of elution buffer. The eluate was concentrated using an ultrafiltration centrifuge tube, and the protein concentration was determined using a BCA Protein Quantification Kit (Tiangen, Beijing, China). The purified recombinant proteins were designated as NA, ALC001, ALC005, and ALC007 and stored at −80 °C for further use.

### 2.4. Determination of Antibacterial Activity of Recombinant Proteins Against Host Bacteria

The antibacterial activities of recombinant proteins (NA, ALC001, ALC005, and ALC007) against *R. anatipestifer* RAf1 were evaluated as follows: The bacterial suspension was diluted 100-fold in 20 mM Tris-HCl (pH 7.0) to a final density of ~10^6^ CFU/mL. For the control group, 100 μL of the diluted suspension was mixed with 100 μL of 20 mM Tris-HCl (pH 7.0). For the experimental groups, 100 μL of the diluted suspension was combined with 100 μL of the recombinant proteins at various concentrations (0.4, 0.6, 0.8, and 1.0 mg/mL) to achieve final concentrations of 0.2, 0.3, 0.4, and 0.5 mg/mL, respectively. After a 2 h incubation, appropriate dilutions of the cell suspensions were plated onto TSA in triplicate. Colonies were counted after overnight incubation at 37 °C. The antibacterial activity was quantified as the relative inactivation in log units, calculated as log_10_(N_0_/N_i_), where N_0_ is the initial number of untreated cells and N_i_ is the number of residual cells counted after treatment [10].

### 2.5. Assessment of Antibacterial Activity of Recombinant Protein ALC005

ALC005 was diluted to a concentration of 0.8 mg/mL. Its antibacterial activity was then evaluated against eighteen *R. anatipestifer* strains, five *E. coli* isolates, and five *Salmonella* strains following the procedure described in Section 2.4. The host strain RA1, which is susceptible to phage CRP2, was included as a positive control. Antibacterial activity is reported as the log_10_ reduction in viable bacterial count.

### 2.6. Influence of Incubation Time on the Antibacterial Activity of ALC005

ALC005 was prepared at a concentration of 0.8 mg/mL. Following the method described in Section 2.4, the antibacterial activity against *R. anatipestifer* RAf1 was assessed at different incubation intervals: 0.5, 1, 2, and 3 h.

### 2.7. Impacts of Temperature and pH on the Antibacterial Activity of ALC005

To evaluate its stability, ALC005 was subjected to varying temperatures and pH levels. For temperature stability, ALC005 (0.8 mg/mL) was incubated in water baths at 0 °C, 40 °C, 50 °C, and 60 °C for 30 min. For pH stability, ALC005 (1.6 mg/mL) was mixed with an equal volume of 20 mM Tris-HCl buffer at various pH levels (5, 6, 7, 8, and 9). The mixtures were incubated in ice water for 60 min. Following each treatment, the residual antibacterial activity against *R. anatipestifer* RAf1 was determined according to the procedure described in Section 2.4.

### 2.8. Morphological Analysis of ALC005-Treated Host Bacteria

A bacterial suspension of *R. anatipestifer* RAf1 (50 μL) was mixed with either PBS (950 μL) or ALC005 (950 μL, 1.6 mg/mL). Following a 3 h incubation at 37 °C, 10 μL of each mixture was applied to copper grids and allowed to adsorb for 15 min. Excess liquid was blotted with filter paper, and the grids were stained with one drop of 20 g/L phosphotungstic acid (pH 6.8) for 5 min. After removing the residual stain, the samples were air-dried and examined using a Hitachi HT7700 (Hitachi High-Tech Corporation, Tokyo, Japan) transmission electron microscope.

### 2.9. Statistical Analysis

All experiments were performed in vitro with three biological replicates. Data are expressed as Mean ± standard deviation (SD). Statistical analyses and graphical presentations were carried out using GraphPad Prism version 10.5.0 (GraphPad Software Inc., La Jolla, CA, USA). For multiple group comparisons, one-way analysis of variance (ANOVA) was applied, followed by Tukey’s honestly significant difference (HSD) post hoc test for pairwise comparisons. Statistical significance was determined at a *p*-value of <0.05 (*), <0.01 (**), <0.001 (***), or <0.0001 (****), while *p*-values exceeding 0.05 were considered not significant (ns).

## 3. Results and Analysis

### 3.1. Purification and Identification of Recombinant Proteins

**Figure 2:**
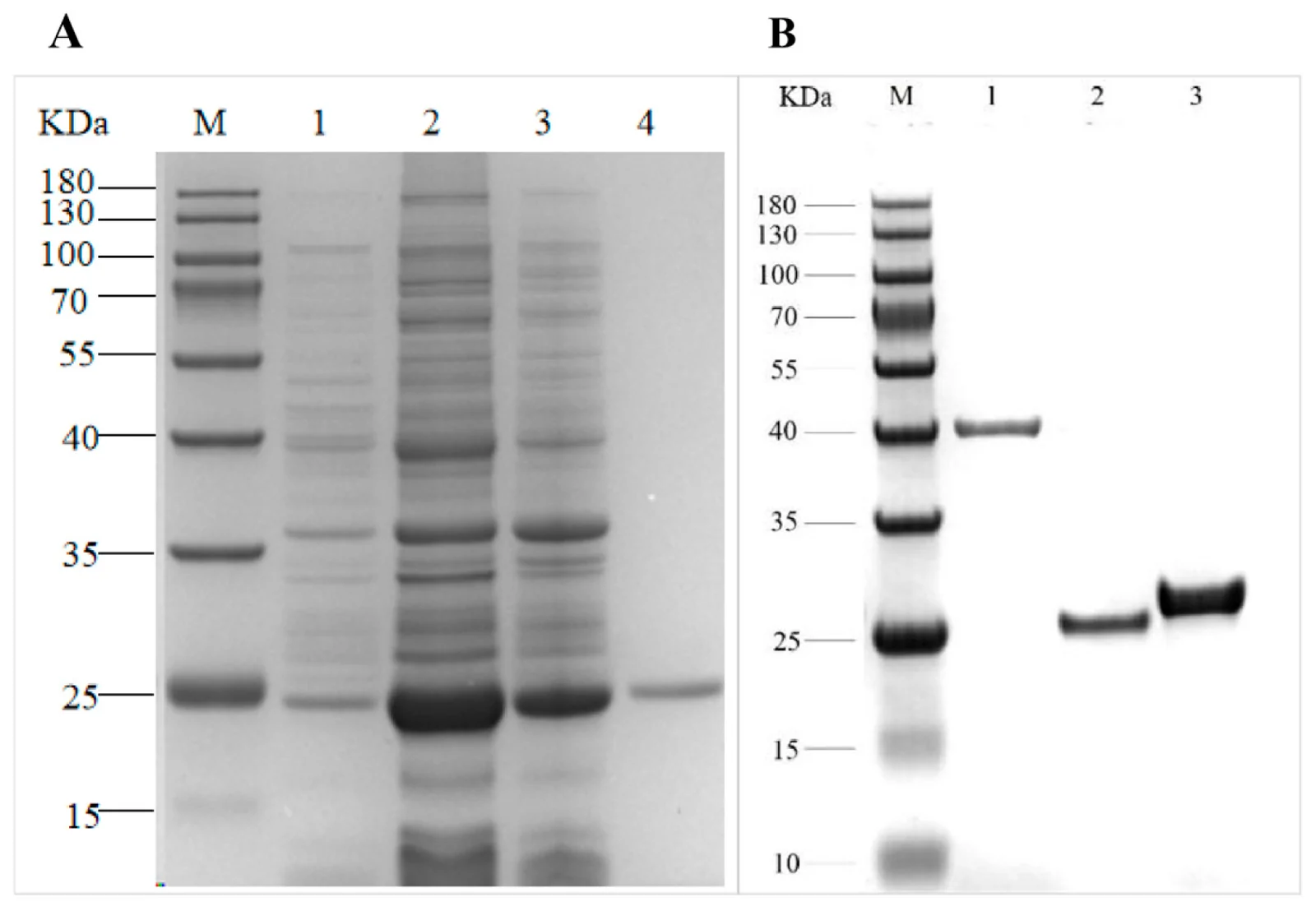
SDS-PAGE results of recombinant proteins NA, ALC001, ALC005, and ALC007. (**A**) SDS-PAGE analysis of recombinant protein NA. M: Protein molecular weight marker; Lane 1: Uninduced bacterial culture; Lane 2: Supernatant of induced bacterial lysate; Lane 3: Pellet of induced bacterial lysate; Lane 4: Purified NA (21.7 kDa). (**B**) SDS-PAGE analysis of recombinant proteins ALC001, ALC005, and ALC007. M: Protein molecular weight marker; Lane 1: Purified ALC001 (46.3 kDa); Lane 2: Purified ALC005 (23.3 kDa); Lane 3: Purified ALC007 (23.1 kDa).

SDS-PAGE analysis was performed to evaluate the expression patterns of the recombinant proteins. For NA, distinct bands corresponding to the expected molecular weight of 21.7 kDa were observed in both the soluble lysate and insoluble pellet fractions, indicating that NA was expressed in a mixed manner—partially in soluble form and partially as inclusion bodies (Figure 2A). Similarly, ALC001, ALC005, and ALC007 exhibited comparable mixed expression profiles, with target proteins detectable in both soluble and insoluble fractions. The observed bands corresponded to their predicted molecular weights of 46.3 kDa (ALC001), 23.3 kDa (ALC005), and 23.1 kDa (ALC007), respectively (Figure 2B). Mass spectrometry analysis of the four recombinant protein target bands further confirmed their identities, with amino acid sequence coverage rates of 100%, 83%, 89%, and 92% relative to the expected sequences. These results demonstrate that the recombinant proteins were correctly expressed and effectively purified.

### 3.2. Antibacterial Activity of NA, ALC001, ALC005, and ALC007

**Figure 3:**
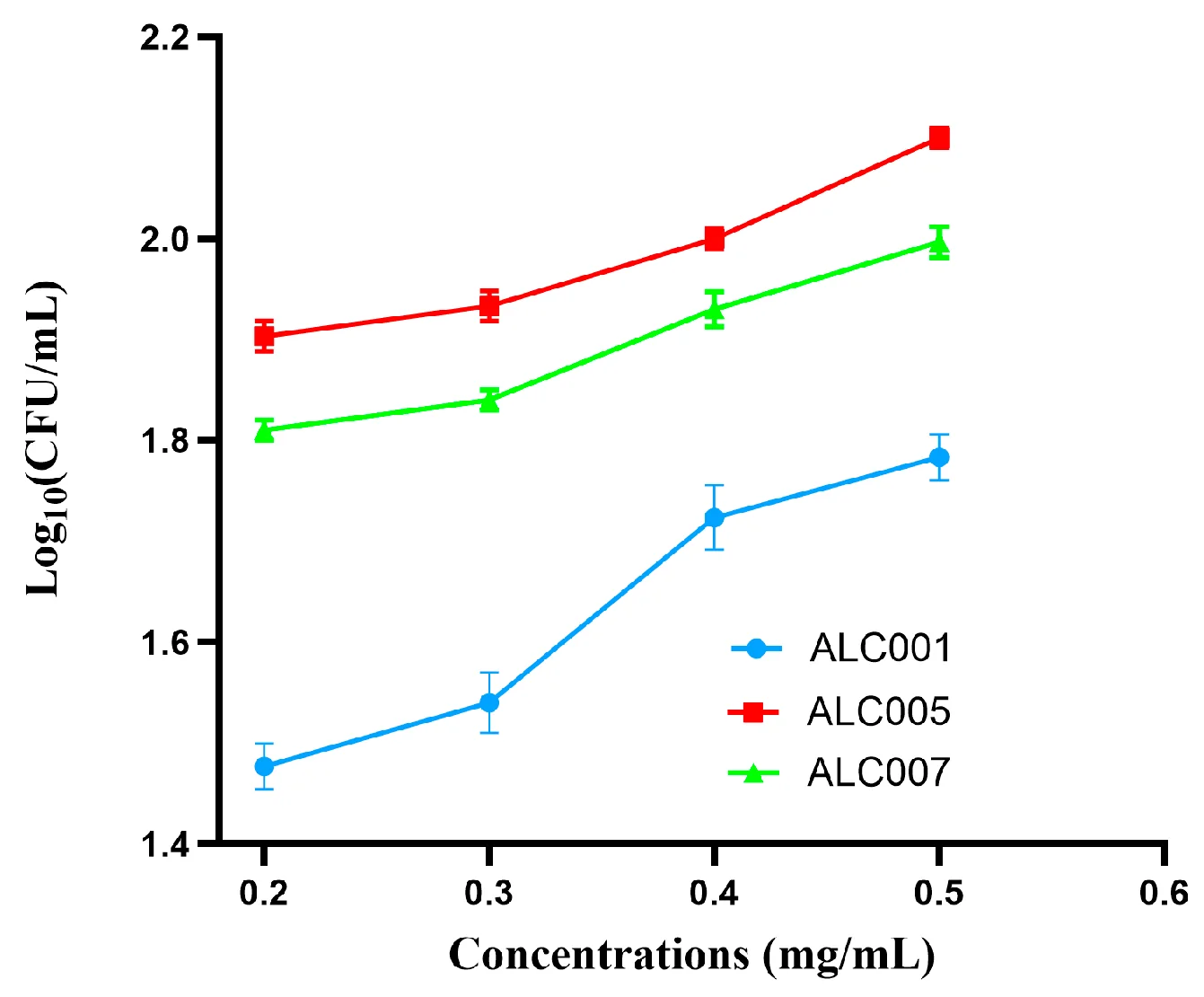
Antibacterial activity of recombinant proteins (ALC001, ALC005, ALC007) ranging from 0.2 to 0.5 mg/mL. The experiments were set with three biological replicates, and the data are presented as Mean ± SD.

The antibacterial activities of the recombinant proteins NA, ALC001, ALC005, and ALC007 against *R. anatipestifer* RAf1 were quantitatively assessed by measuring the log_10_ reduction values across a concentrations range of 0.2–0.5 mg/mL (Figure 3). NA exhibited no detectable antibacterial effect, as indicated by the absence of a significant difference in viable bacterial counts between the treatment and control groups. In contrast, ALC001, ALC005, and ALC007 induced a dose-dependent increase in antibacterial activity. Among these, ALC005 consistently demonstrated the highest antibacterial activity across the entire concentration range. Its dose–response curve was positioned distinctly above those of the other proteins, initiating at a log_10_ reduction of 1.90 ± 0.02 log units at 0.2 mg/mL and reaching a maximum of 2.10 ± 0.01 log units at 0.5 mg/mL. This value was significantly higher than those of ALC007 (2.00 ± 0.02 log units) and ALC001 (1.78 ± 0.02 log units). Notably, even at the lowest tested concentration (0.2 mg/mL), the antibacterial activity of ALC005 (1.90 ± 0.02 log units) surpassed that of ALC001 at its maximum concentration of 0.5 mg/mL (1.78 ± 0.02 log units).

### 3.3. Strain-Specific Bacteriolytic Activity of ALC005

**Figure 4:**
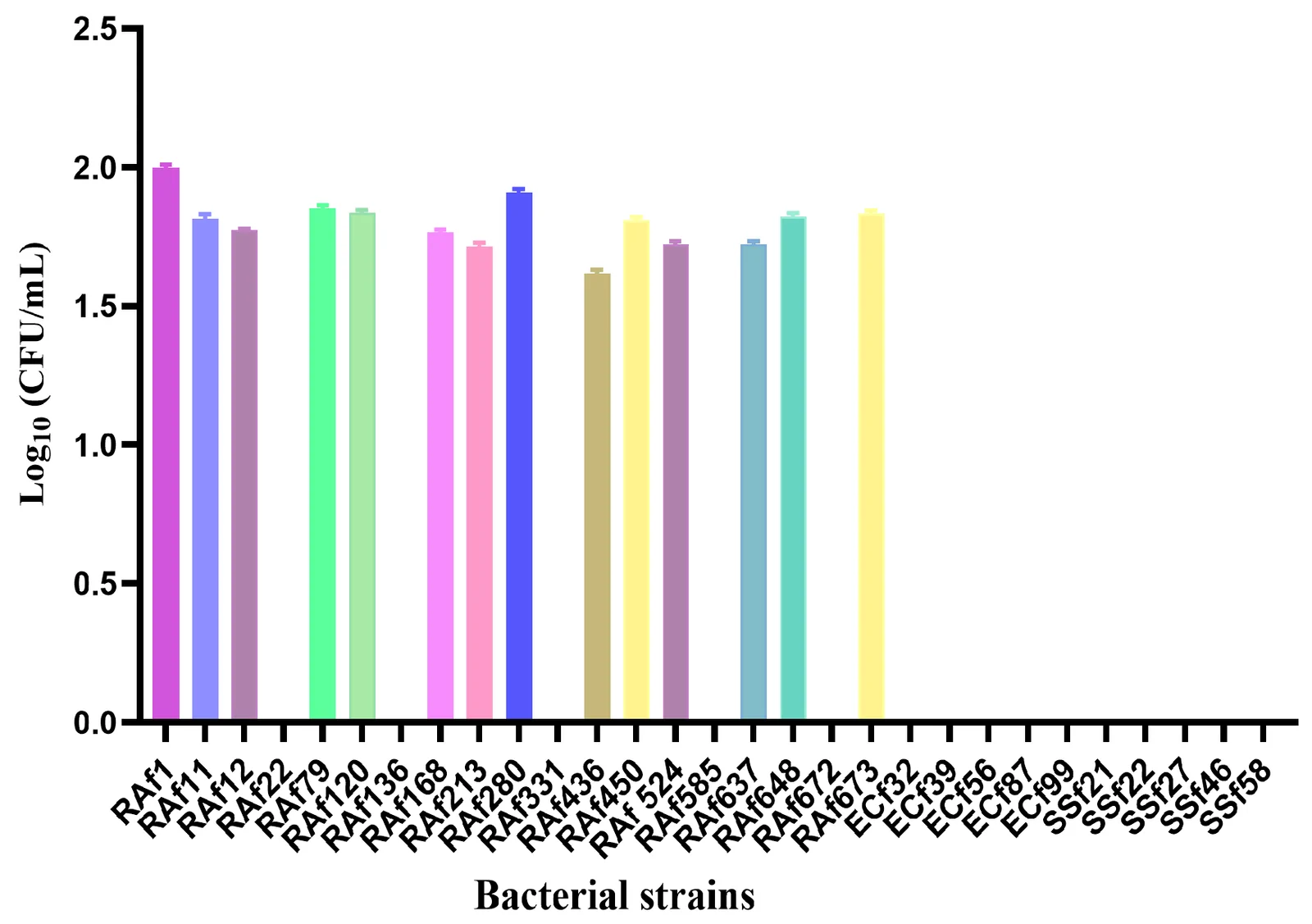
The lysis spectrum of ALC005. The experiments were set with three biological replicates, and the data are presented as Mean ± SD.

The bacteriolytic spectrum of ALC005 was determined against a panel of 28 bacterial isolates, comprising 18 *R. anatipestifer* strains, 5 *E. coli* strains, and 5 *Salmonella* strains (Figure 4). The host strain RA1, which is susceptible to phage CRP2, was included as a positive control. ALC005 exhibited potent lytic activity against the positive control strain RA1, confirming the bioactivity of the recombinant protein. Notably, ALC005 induced a substantial reduction (>1.6 log units) in 13 of the 18 *R. anatipestifer* strains tested, with a lysis rate of 72.2%. In contrast, no measurable reduction was observed against the remaining 5 *R. anatipestifer* strains. Furthermore, ALC005 displayed no detectable lytic activity against any of the 5 *E. coli* strains or 5 *Salmonella* strains. Collectively, these data indicate that ALC005 possesses a distinct strain-selective antibacterial profile, demonstrating favorable activity against specific *R. anatipestifer* strains but being ineffective against the *E. coli* and *Salmonella* strains tested.

### 3.4. Effect of Incubation Time on the Antibacterial Activity of ALC005

**Figure 5:**
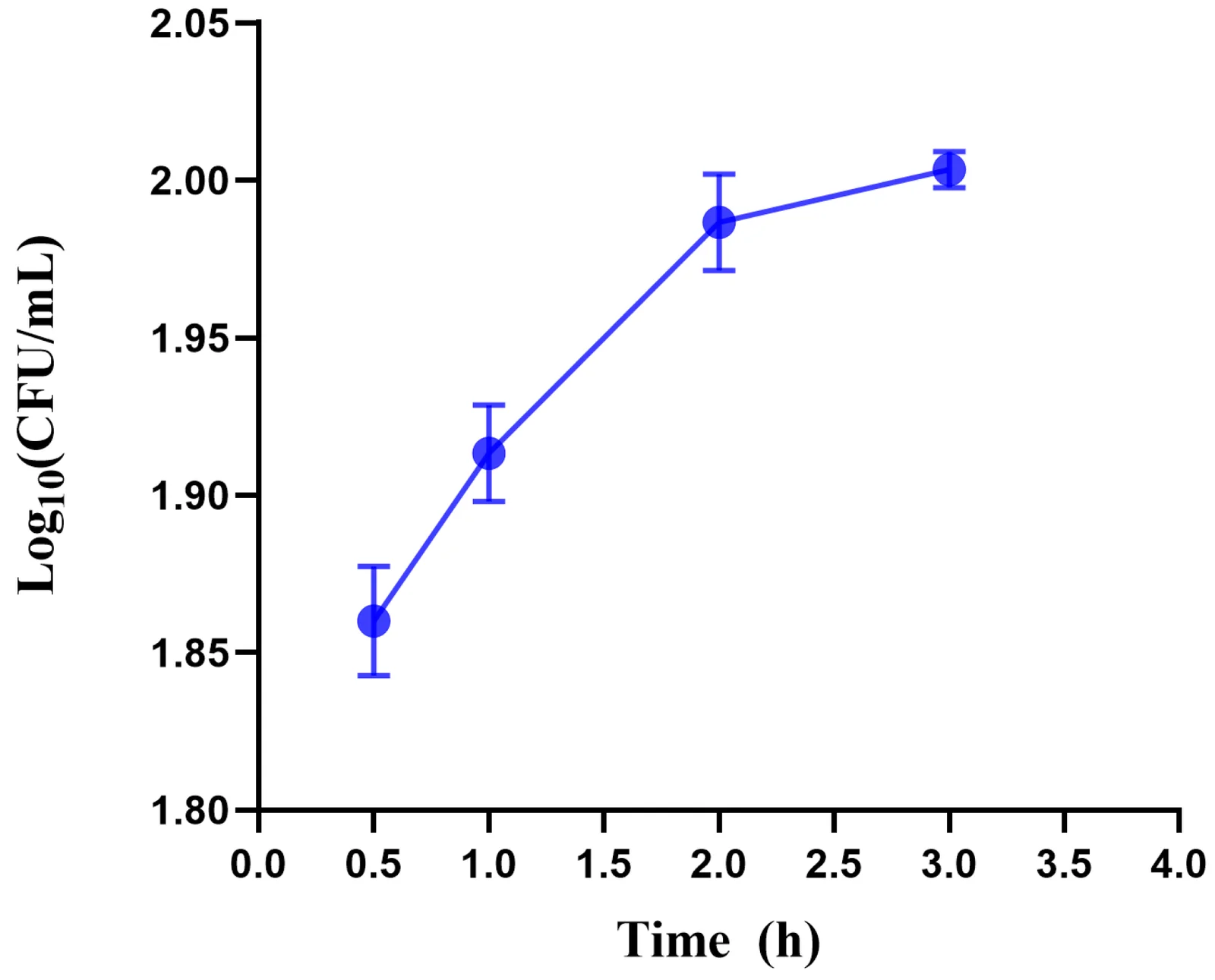
Results of the effect of exposure time on the antibacterial activity of ALC005. The experiments were set with three biological replicates, and the data are presented as Mean ± SD.

The antibacterial activity of ALC005 against RAf1 was quantitatively evaluated over a 3 h period by monitoring the log_10_ reduction in bacterial viability relative to an untreated control (Figure 5). Analysis of the time-killing curve revealed a distinct temporal progression in the bactericidal activity of ALC005. During the initial 0.5 h exposure, ALC005 induced a measurable log_10_ reduction of 1.86 ± 0.02 log units, confirming its immediate bioactivity. The antibacterial effect intensified progressively over time, with a substantial reduction of 1.91 ± 0.02 log units observed at the 1 h time point. This time-dependent enhancement continued through the 2 h exposure, demonstrating consistently strengthened lytic capacity. The maximum antibacterial efficacy was achieved at the 3 h time point, where the log_10_ reduction reached its peak value of 2.00 ± 0.01 log units, representing the most potent lytic activity observed during the experimental period. These findings establish that the antibacterial efficacy of ALC005 against RAf1 is both immediate and progressively strengthening over time, as reflected by the increasing log_10_ reduction values.

### 3.5. Effect of Temperature on the Antibacterial Activity of ALC005

**Figure 6:**
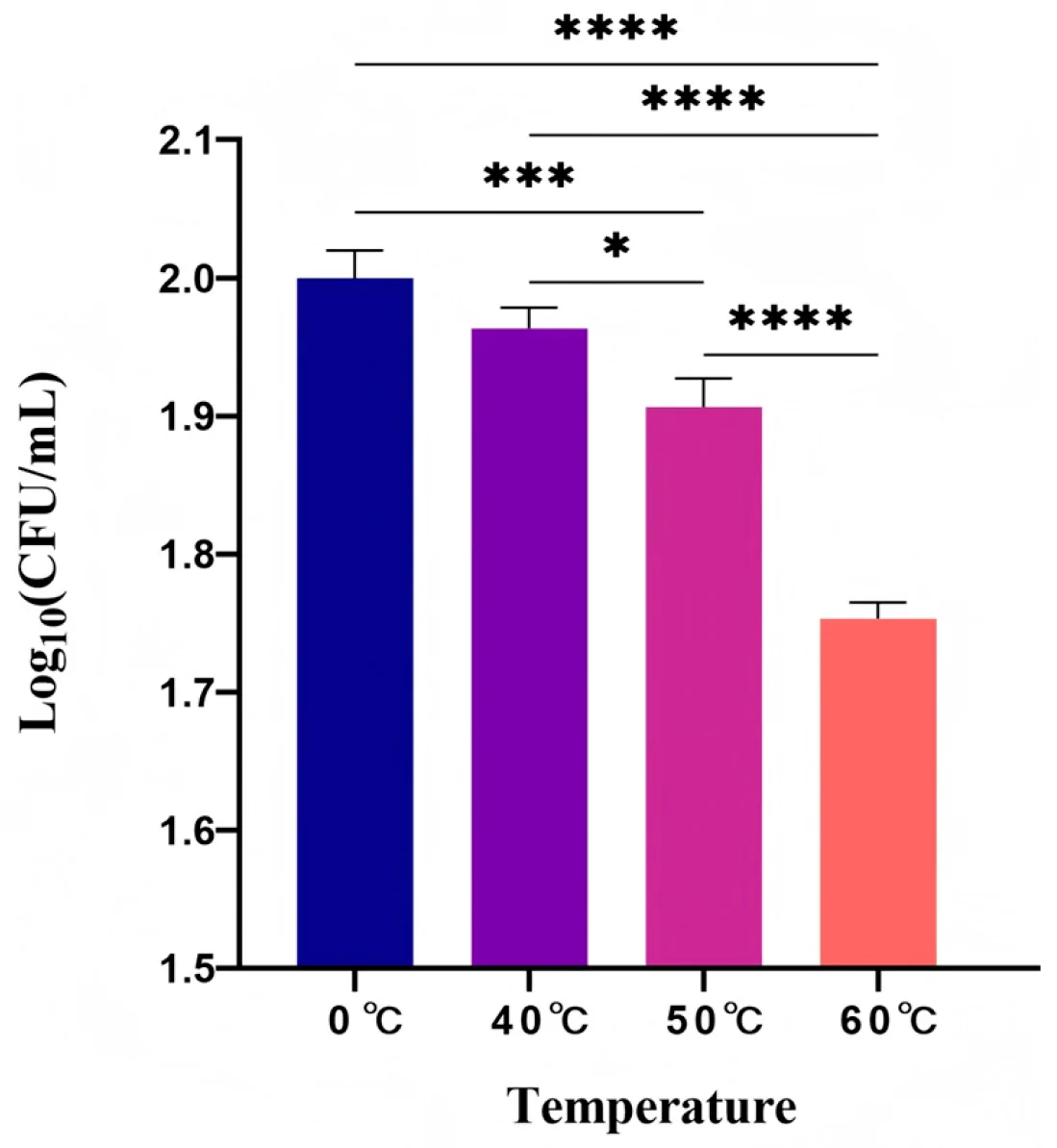
Results of thermal stability test of ALC005. The experiments were set with three biological replicates, and the data are presented as Mean ± SD. *p* values were determined by One-way analysis of variance with Tukey’s multiple comparisons test as a post-hoc test. Statistical significance was determined at a *p*-value of <0.05 (*), <0.001 (***), or <0.0001 (****).

The thermostability of ALC005 was evaluated by measuring its residual antibacterial activity after incubation at different temperatures. As shown in Figure 6, ALC005 exhibited high antibacterial activity with the reductions of 2.00 ± 0.02 and 1.96 ± 0.02 log units at 0 °C and 40 °C, respectively. However, a significant decrease in activity was observed at 50 °C (1.91 ± 0.02 log units) compared to both lower temperature groups (*p* < 0.05). The most substantial reduction in lytic efficacy occurred at 60 °C (1.75 ± 0.01 log units), which was statistically significant compared to all other temperature conditions tested (*p* < 0.01). These results demonstrate that ALC005 retains substantial bacteriolytic activity within a moderate temperature range (0–40 °C) without significant loss of efficacy. The protein begins to show significant thermal instability at temperatures above 50 °C, with marked reduction observed at 60 °C. This thermostability profile suggests that ALC005 maintains functional integrity under standard storage and application temperatures.

### 3.6. Effect of pH on the Antibacterial Activity of ALC005

**Figure 7:**
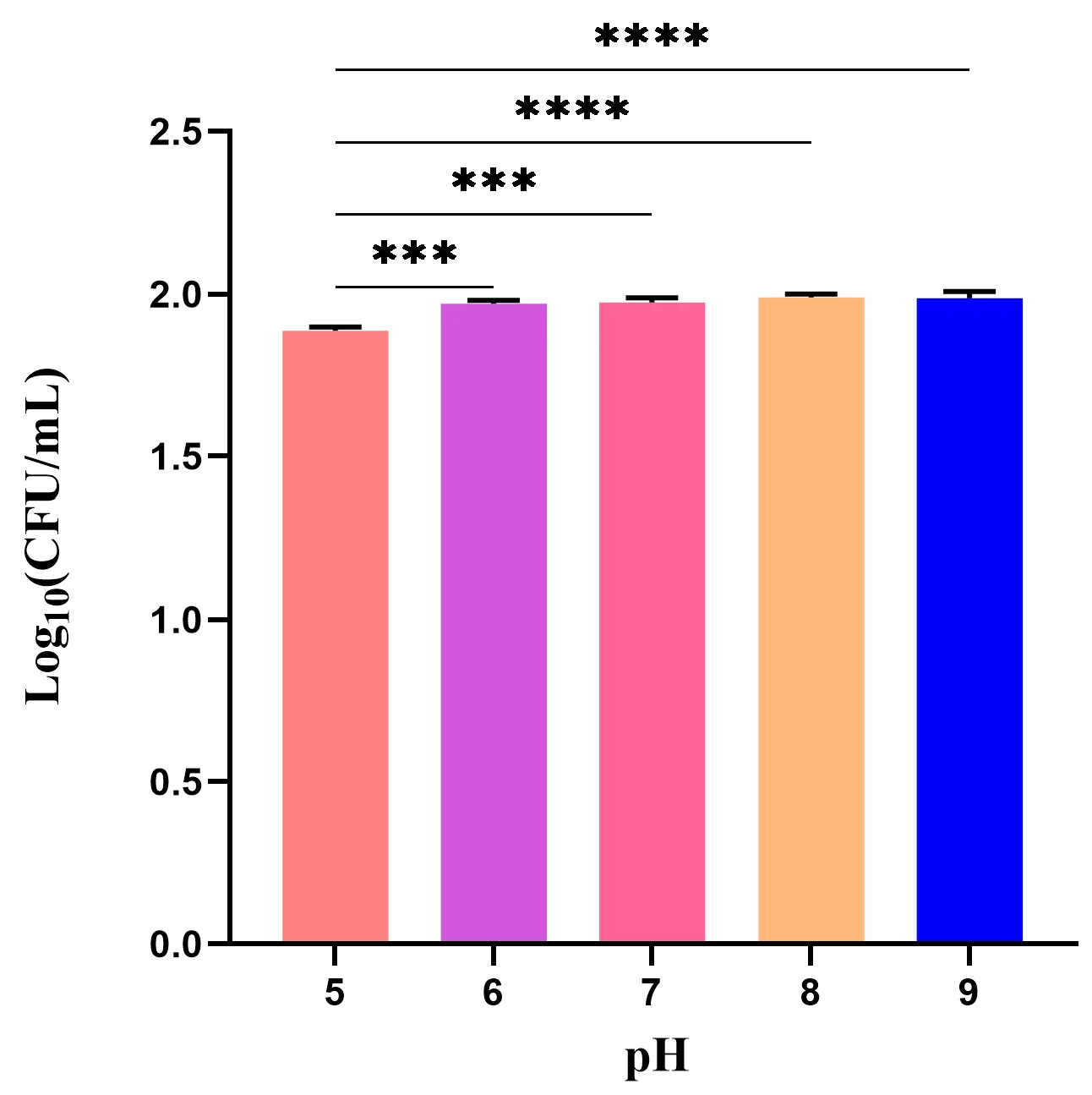
Results of pH stability test of ALC005. The experiment was set up with three biological replicates, and the data are presented as Mean ± SD. *p* values were determined by One-way analysis of variance with Tukey’s multiple comparisons test as a post-hoc test. Statistical significance was determined at a *p*-value of <0.001 (***) or <0.0001 (****).

To assess the stability and functional range of ALC005, its lytic activity was evaluated under different pH conditions (Figure 7). The activity at pH5 was significantly lower than that at all other pH conditions tested, with a log_10_ reduction of 1.89 ± 0.01 log units (*p* < 0.05). No statistically significant difference (*p* > 0.05) was found between the lytic activities at pH 6–9. This indicates that the protein’s performance stabilizes and remains at a similarly high level across this range. The protein exhibits optimal and statistically equivalent performance at pH 6–9, with a noticeable but modest decrease in activity only under more acidic conditions (pH 5). This broad pH stability profile underscores the robustness of ALC005 and suggests its potential suitability for applications in diverse physiological environments.

### 3.7. Morphological Changes in Cells Following ALC005 Treatment

**Figure 8:**
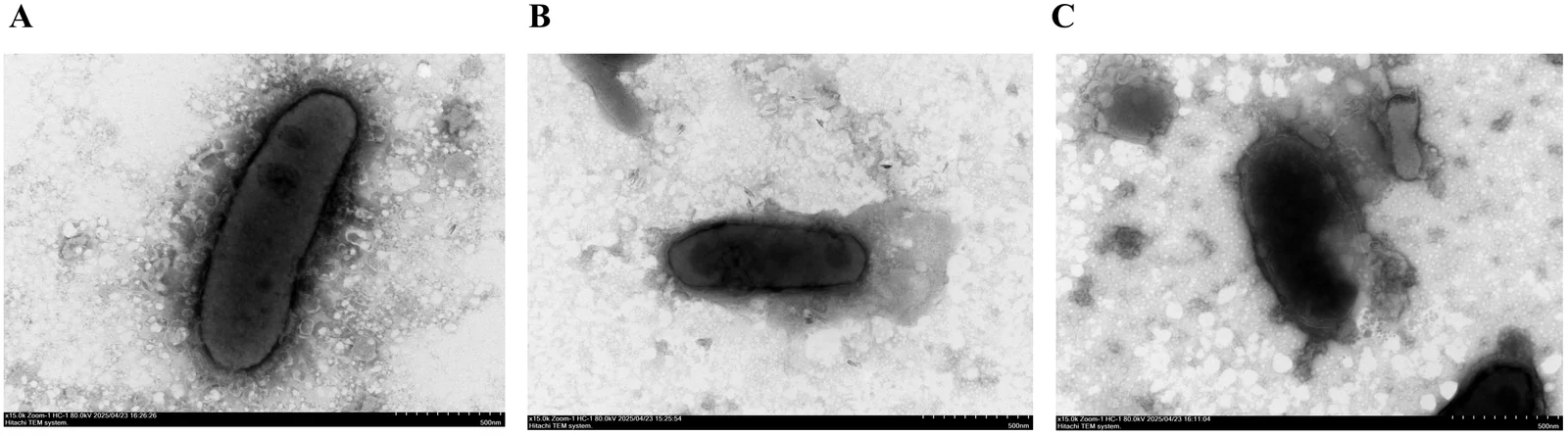
Morphology of bacteria after ALC005 treatment. (**A**): Untreated bacteria are intact; (**B**): Treated bacteria show slight damage with cytoplasmic leakage; (**C**): Treated bacteria are lysed, with cellular debris released.

The structural integrity of RAf1 cells after exposure to ALC005 was examined using transmission electron microscopy (TEM). As demonstrated in Figure 8, treatment with ALC005 at a concentration of 1.6 mg/mL induced severe morphological alterations in the bacterial cells. Compared to the intact structure of untreated controls (Figure 8A), the treated cells exhibited dramatic disruption of the cell wall and membrane integrity (Figure 8B,C). This damage was characterized by the apparent lysis of the cellular envelope and the consequent release of cytoplasmic contents into the extracellular environment. These ultrastructural observations confirm that ALC005 exerts a direct and destructive effect on the cell envelope of RAf1, leading to bacteriolysis.

## 4. Discussion

Due to its complex serotypes and increasingly severe multidrug resistance, *R. anatipestifer* has become a significant threat to poultry farming, creating an urgent need for novel therapeutic agents against this pathogen [8]. Phage-encoded endolysins have emerged as a research focus in antimicrobial therapy due to their rapid action, high specificity, and low potential for inducing resistance [31]. However, the outer membrane structure unique to Gram-negative bacteria effectively blocks endolysins from accessing their peptidoglycan substrates, severely limiting their bactericidal efficacy [32]. In this study, the natural endolysin NA derived from *R. anatipestifer* phage CRP2 showed no antibacterial activity. This result is consistent with recent findings on the *R. anatipestifer* endolysin LysGDF21, which also lacks direct antibacterial activity and requires an outer membrane permeabilizer to exert its lytic function [18]. To overcome the outer membrane barrier of Gram-negative bacteria, various strategies have been employed to enhance the outer membrane penetration ability of endolysins, including fusion with phage RBPs or CPPs, introduction of hydrophobic groups or positively charged residues, and domain swapping [33,34,35,36]. To enable the natural endolysin NA to autonomously cross the outer membrane barrier of Gram-negative bacteria, this study incorporated functional outer membrane-penetrating elements based on the above findings.

The first strategy involved fusing the endolysin NA with the RBP. Phage RBPs mediate the recognition and adsorption of phages by binding to specific surface receptors on host bacteria, thereby facilitating precise targeting of the endolysin. Zampara et al. fused the endolysin LysEC8 with the RBP Pb5 from phage T5, and the resulting recombinant protein reduced *E. coli* load by 2.2 log units [37]. Subsequently, the same team fused the tail fiber protein H encoded by a *Campylobacter jejuni* (*C. jejuni*) prophage with the T5 endolysin, achieving effective killing of *C. jejuni* [38]. Previous studies have shown that the tail fiber protein encoded by *R. anatipestifer* phage CRP2 itself functions as a RBP, with its N-terminal region being relatively conserved across different strains [30]. Based on these observations, the conserved N-terminal region of this RBP was fused to NA to generate ALC001.

The second strategy involved fusing the endolysin NA with the CPP. Fusing CPPs to endolysins has been demonstrated to significantly enhance their bactericidal activity against Gram-negative bacteria. Lim et al. fused the CPP DS4.3 with endolysin PA90 to generate DS-PA90, which completely killed *Acinetobacter baumannii* at a concentration of 0.25 μM and exhibited strong in vivo protective effects in a wax moth larva infection model [39]. Consistently, similar CPP fusion strategies have been successfully applied to other endolysin systems. For instance, Abdelkader et al. engineered a chimeric endolysin, eLysMK34, by fusing the antimicrobial peptide cecropin A to the N-terminus of LysMK34, which significantly enhanced its activity against colistin-resistant *Acinetobacter baumannii* strains, achieving MIC values as low as 0.45 μM [40]. Accordingly, the CPP was incorporated into NA to construct ALC005.

The third strategy involved fusing the endolysin NA with the PCNP. The PCNP fusion strategy has also made significant progress. The representative Artilysin Art-175, developed by Briers et al., reduced viable counts of *Pseudomonas aeruginosa* and *Acinetobacter baumannii* by 4–5 log units within 30 min [41]. Its optimized homolog Art-085, which exhibits enhanced thermostability, demonstrated comparable bactericidal efficacy [26]. These precedents support the fusion of the PCNP to the endolysin NA, resulting in ALC007.

In this study, the C-terminus of NA was separately fused with the phage RBP, CPP, and PCNP to systematically compare the relative effectiveness of these three strategies in enhancing antibacterial activity against *R. anatipestifer*. Antimicrobial activity assays revealed that all three modified proteins—ALC001, ALC005, and ALC007—exhibited clear dose-dependent effects. Among them, ALC005, which incorporated the CPP, demonstrated the strongest activity, significantly outperforming both ALC007 and ALC001. Specifically, at a concentration of 0.5 mg/mL, ALC005 reduced the RAf1 bacterial load by 2.10 ± 0.01 log units, an efficacy comparable to that of other engineered endolysins targeting Gram-negative bacteria reported previously [21,37]. Notably, ALC005 achieved a 1.90 ± 0.02 log_10_ reduction at a low concentration of 0.2 mg/mL, surpassing the bactericidal effect of ALC001 at 0.5 mg/mL (1.78 ± 0.02 log units), further highlighting the superior antibacterial efficacy of ALC005. These results suggest that the non-specific outer membrane penetration capability conferred by the CPP may represent a more effective pathway for enhancing the bactericidal activity of the *R. anatipestifer* endolysin NA.

ALC005 exhibited clear strain selectivity: among 18 tested *R. anatipestifer* strains, 13 showed a significant reduction in bacterial load (>1.6 log units), while 5 showed no response. Furthermore, no detectable lytic activity was observed against *E. coli* or *Salmonella*. Phages and their endolysins typically display species-specific activity [42]. A similar phenomenon has also been reported for the engineered endolysin Art-175, which demonstrated strong bactericidal activity against *Acinetobacter baumannii* and *Pseudomonas aeruginosa* but weak activity against *E. coli* and *Salmonella* [41]. Although this selectivity somewhat limits its potential as a broad-spectrum antimicrobial agent, from the perspective of microbiome safety, such targeted specificity helps minimize disruption to commensal microbiota during treatment and reduces off-target risks.

The time-killing curve showed that ALC005 induced a significant reduction in RAf1 bacterial load within 0.5 h (1.86 ± 0.02 log units), with the bactericidal effect continuously increasing over time and reaching its peak at 3 h (2.00 ± 0.01 log units). Similarly, several engineered endolysins have been reported to rapidly kill target bacteria within minutes [26], indicating that the CPP fusion strategy offers broad advantages in achieving rapid bactericidal activity, which is crucial for controlling acute infections.

Temperature and pH stability tests revealed that ALC005 maintained stable activity across a range of 0–40 °C, with significant loss of activity observed above 50 °C and nearly complete inactivation at 60 °C. Activity remained largely unchanged between pH 6 and 9, with only a slight reduction at pH 5. These characteristics are consistent with most reported Gram-negative-targeting endolysins [43,44], suggesting that ALC005 possesses excellent environmental tolerance. This makes it particularly suitable for application in neutral to weakly alkaline physiological microenvironments, such as the duck intestine.

TEM observations directly confirmed the bactericidal mechanism of ALC005 at the morphological level. After treatment with ALC005, RAf1 cells exhibited significant disruption of cell wall and cell membrane structures, leading to leakage of intracellular contents and ultimately complete lysis of the bacterial cells [45].

In summary, this study systematically evaluated three fusion strategies—RBP, CPP, and PCNP—and identified CPP fusion as the most effective approach for enhancing the antibacterial activity of endolysin NA against *R. anatipestifer*. The resulting novel engineered endolysin, ALC005, demonstrated rapid bactericidal action, satisfactory stability under mild conditions, and moderate strain selectivity. While these in vitro findings provide a theoretical foundation and experimental insights for the rational design of engineered endolysins, we acknowledge that in vivo validation in duck models is warranted to further evaluate its therapeutic potential and safety. Future studies will address this limitation by conducting systematic in vivo efficacy and safety assessments.

## 5. Conclusions

CPP fusion was identified as the optimal strategy for potentiating the antibacterial efficacy of endolysin NA against *R. anatipestifer*. Notably, ALC005, the CPP-fused chimera, outperformed the other constructs, exhibiting rapid bactericidal kinetics, favorable stability, and moderate strain specificity. These attributes collectively underscore the potential of ALC005 and provide a robust experimental foundation for guiding future engineering and optimization of endolysin-based agents.

## Figures and Tables

**Table 1:** Spectrogram determination of ALC005.

Bacteria	Strains	Cleavage by ALC005
*R. anatipestifer*	RAf1(Host Strain)	Yes
	RAf11	Yes
	RAf12	Yes
	RAf22	Yes
	RAf79	No
	RAf120	Yes
	RAf136	No
	RAf168	Yes
	RAf213	Yes
	RAf280	Yes
	RAf331	No
	RAf436	Yes
	RAf450	Yes
	RAf524	Yes
	RAf585	No
	RAf637	Yes
	RAf648	Yes
	RAf672	No
	RAf673	Yes
*E. coli*	ECf32	No
	ECf39	No
	ECf56	No
	ECf87	No
	ECf99	No
*Salmonella*	SSf21	No
	SSf22	No
	SSf27	No
	SSf46	No
	SSf58	No

## Data Availability

The raw data supporting the conclusions of this article will be made available by the authors on request.

## Abbreviations

The following abbreviations are used in this manuscript:

*R. anatipestifer*	*Riemerella anatipestifer*
LB	Lysogeny Broth
RBP	receptor-binding protein
CPP	cell-penetrating peptide
PCNP	polycationic nonapeptide
TSA	Tryptic Soy Agar
TSB	Tryptic Soy Broth
IPTG	isopropyl-β-D-thiogalactopyranoside
CRP2	vB_RanS_CRP2
SD	standard deviation
OD	Optical density
CFU	Colony-forming unit
h	hour
TEM	Transmission electron microscopy

## References

[1] Hao J., Zhang J., He X., Wang Y., Su J., Long J., Zhang L., Guo Z., Zheng Y., Wang M. (2025). Unveiling the silent threat: A comprehensive review of *Riemerella anatipestifer*—From pathogenesis to drug resistance. Poult. Sci..

[2] Zhang C., Liu D., Sui Z., E W., Gogoi-Tiwari J., Liu H., Sun Y., Yang S., Cui A., Liu C. (2025). Epidemiological investigation of *Riemerella anatipestifer* in large-scale chicken farms in 29 provinces of China from 2021 to 2024. Poult. Sci..

[3] Zhang Y., Wang X., Wang Y., Sun J., Dong W., Meng K., Li G., Yuan X. (2025). Biological and genomic characteristics of chicken-derived *Riemerella anatipestifer* in China. Front. Microbiol..

[4] Zhou W., Cui X., Zhou S., Liu S., Peng C., Yang J., Li J., Hou G., Jiang W., Liu H. (2025). Spillover of *Riemerella anatipestifer* to laying hens leads to a decrease in egg production and hatchability. Poult. Sci..

[5] Guo F., Shi C., Huang L., Yao Y., Qi Y., Zhu D., Wang M., Jia R., Chen S., Zhao X. (2025). Functional characterization of the chaperone DnaK of *Riemerella anatipestifer* in antibiotic resistance and pathogenicity. Poult. Sci..

[6] Liu Y., Luo S., Yang Z., Wang M., Jia R., Chen S., Liu M., Zhao X., Yang Q., Wu Y. (2023). Capsular polysaccharide determines the serotyping of *Riemerella anatipestifer*. Microbiol. Spectr..

[7] Lan J., Wu S., Zhao L., Li F., Xing D., Li F., Tian H., Yang X., Sun S., Wang M. (2025). Research Progress in the Development of Vaccines Against *Riemerella anatipestifer*. Microorganisms.

[8] Li Y., Huang Z., Zhu X., Liu C., Cao S., Li Y. (2025). Serotyping, molecular typing, and vaccine protein screening for *Riemerella anatipestifer*: Overcoming challenges in prevention and treatment. Vet. Microbiol..

[9] Aitken M., Abeysekera G., Billington C., Dobson R.C.J. (2025). Recent Advances in Endolysin Engineering. Antibiotics.

[10] Ma W., Lei Y., Wang L., Chen Y., Ma M., Chen X. (2026). Bacteriophage Lysins as Programmable Antimicrobials: Mechanisms, Engineering, and Translational Advances. Probiot. Antimicrob. Proteins.

[11] Qian H., Zheng Y. (2026). Phage endolysins as alternative antimicrobials: Mechanisms, clinical progress, and emerging resistance frameworks. Front. Microbiol..

[12] Sabur A., Khan A., Borphukan B., Razzak A., Salimullah M., Khatun M. (2025). The Unique Capability of Endolysin to Tackle Antibiotic Resistance: Cracking the Barrier. J. Xenobiot..

[13] Pastagia M., Schuch R., Fischetti V.A., Huang D.B. (2013). Lysins: The arrival of pathogen-directed anti-infectives. J. Med. Microbiol..

[14] Ahmadbeigi Y., Soleimani N., Azizmohseni F., Amini-Bayat Z. (2025). ZAM-CS, a novel chimeric endolysin with enhanced stability and rapid action against methicillin-resistant Staphylococcus aureus. BMC Microbiol..

[15] N H., Saha A., Mandal A., Rout S., More S.S., Mitra S.D. (2026). Combination of endolysin LysK04 and cinnamaldehyde potentially inhibits Gram-negative pathogens via outer membrane disruption and peptidoglycan hydrolysis. Microb. Pathog..

[16] Sisson H.M., Jackson S.A., Fagerlund R.D., Warring S.L., Fineran P.C. (2024). Gram-negative endolysins: Overcoming the outer membrane obstacle. Curr. Opin. Microbiol..

[17] Kim J., Son S.M., Ahn E., Park H., Ryu S. (2025). Surface charge of the C-terminal helix is crucial for antibacterial activity of endolysin against Gram-negative bacteria. J. Biomed. Sci..

[18] Zhao Y., Liu Y., Jiang Y., Li X., Si Z., Lu J., Cao S., Xue X., Li Y., Liu C. (2025). Characterization of a new lytic bacteriophage vB_RanS_GDF21 and its endolysin LysGDF21 with antimicrobial activity against *Riemerella anatipestifer*. Front. Microbiol..

[19] Oliveira H., Thiagarajan V., Walmagh M., Sillankorva S., Lavigne R., Neves-Petersen M.T., Kluskens L.D., Azeredo J. (2014). A thermostable Salmonella phage endolysin, Lys68, with broad bactericidal properties against gram-negative pathogens in presence of weak acids. PLoS ONE.

[20] Sisson H.M., Fagerlund R.D., Jackson S.A., Briers Y., Warring S.L., Fineran P.C. (2024). Antibacterial synergy between a phage endolysin and citric acid against the Gram-negative kiwifruit pathogen *Pseudomonas syringae* pv. *actinidiae*. Appl. Environ. Microbiol..

[21] Wojciechowska M. (2025). Endolysins and membrane-active peptides: Innovative engineering strategies against gram-negative bacteria. Front. Microbiol..

[22] Park W., Park M., Chun J., Hwang J., Kim S., Choi N., Kim S.M., Kim S., Jung S., Ko K.S. (2024). Delivery of endolysin across outer membrane of Gram-negative bacteria using translocation domain of botulinum neurotoxin. Int. J. Antimicrob. Agents.

[23] Heselpoth R.D., Euler C.W., Schuch R., Fischetti V.A. (2019). Lysocins: Bioengineered Antimicrobials That Deliver Lysins across the Outer Membrane of Gram-Negative Bacteria. Antimicrob. Agents Chemother..

[24] Xu D., Zhao S., Dou J., Xu X., Zhi Y., Wen L. (2021). Engineered endolysin-based “artilysins” for controlling the gram-negative pathogen Helicobacter pylori. AMB Express.

[25] Briers Y., Schmelcher M., Loessner M.J., Hendrix J., Engelborghs Y., Volckaert G., Lavigne R. (2009). The high-affinity peptidoglycan binding domain of Pseudomonas phage endolysin KZ144. Biochem. Biophys. Res. Commun..

[26] Briers Y., Walmagh M., Van Puyenbroeck V., Cornelissen A., Cenens W., Aertsen A., Oliveira H., Azeredo J., Verween G., Pirnay J.P. (2014). Engineered endolysin-based “Artilysins” to combat multidrug-resistant gram-negative pathogens. mBio.

[27] Ma Q., Guo Z., Gao C., Zhu R., Wang S., Yu L., Qin W., Xia X., Gu J., Yan G. (2017). Enhancement of the direct antimicrobial activity of Lysep3 against Escherichia coli by inserting cationic peptides into its C terminus. Antonie Van Leeuwenhoek.

[28] Gerstmans H., Criel B., Briers Y. (2018). Synthetic biology of modular endolysins. Biotechnol. Adv..

[29] Sauve K., Watson A., Oh J.T., Swift S., Vila-Farres X., Abdelhady W., Xiong Y.Q., LeHoux D., Woodnutt G., Bayer A.S. (2024). The Engineered Lysin CF-370 Is Active Against Antibiotic-Resistant Gram-Negative Pathogens In Vitro and Synergizes with Meropenem in Experimental Pseudomonas aeruginosa Pneumonia. J. Infect. Dis..

[30] Liu N., Huang Y., Chen H.M., Jiang N.S., Liu R.C., Fu G.H., Fu Q.L., Liang Q.Z., Wan C.H., Li A. (2024). Role of tail fiber protein in phage adsorption to *Riemerella anatipestifer*. Fujian J. Agric. Sci..

[31] Schmelcher M., Loessner M.J. (2016). Bacteriophage endolysins: Applications for food safety. Curr. Opin. Biotechnol..

[32] Briers Y., Lavigne R. (2015). Breaking barriers: Expansion of the use of endolysins as novel antibacterials against Gram-negative bacteria. Future Microbiol..

[33] Gerstmans H., Rodriguez-Rubio L., Lavigne R., Briers Y. (2016). From endolysins to Artilysin(R)s: Novel enzyme-based approaches to kill drug-resistant bacteria. Biochem. Soc. Trans..

[34] Yang H., Yu J., Wei H. (2014). Engineered bacteriophage lysins as novel anti-infectives. Front. Microbiol..

[35] Defraine V., Schuermans J., Grymonprez B., Govers S.K., Aertsen A., Fauvart M., Michiels J., Lavigne R., Briers Y. (2016). Efficacy of Artilysin Art-175 against Resistant and Persistent Acinetobacter baumannii. Antimicrob. Agents Chemother..

[36] Briers Y., Walmagh M., Lavigne R. (2011). Use of bacteriophage endolysin EL188 and outer membrane permeabilizers against Pseudomonas aeruginosa. J. Appl. Microbiol..

[37] Zampara A., Sorensen M.C.H., Grimon D., Antenucci F., Vitt A.R., Bortolaia V., Briers Y., Brondsted L. (2020). Exploiting phage receptor binding proteins to enable endolysins to kill Gram-negative bacteria. Sci. Rep..

[38] Zampara A., Sorensen M.C.H., Gencay Y.E., Grimon D., Kristiansen S.H., Jorgensen L.S., Kristensen J.R., Briers Y., Elsser-Gravesen A., Brondsted L. (2021). Developing Innolysins Against Campylobacter jejuni Using a Novel Prophage Receptor-Binding Protein. Front. Microbiol..

[39] Lim J., Jang J., Myung H., Song M. (2022). Eradication of drug-resistant Acinetobacter baumannii by cell-penetrating peptide fused endolysin. J. Microbiol..

[40] Abdelkader K., Gutierrez D., Tames-Caunedo H., Ruas-Madiedo P., Safaan A., Khairalla A.S., Gaber Y., Dishisha T., Briers Y. (2022). Engineering a Lysin with Intrinsic Antibacterial Activity (LysMK34) by Cecropin A Fusion Enhances Its Antibacterial Properties against Acinetobacter baumannii. Appl. Environ. Microbiol..

[41] Briers Y., Walmagh M., Grymonprez B., Biebl M., Pirnay J.P., Defraine V., Michiels J., Cenens W., Aertsen A., Miller S. (2014). Art-175 is a highly efficient antibacterial against multidrug-resistant strains and persisters of Pseudomonas aeruginosa. Antimicrob. Agents Chemother..

[42] Gondil V.S., Harjai K., Chhibber S. (2020). Endolysins as emerging alternative therapeutic agents to counter drug-resistant infections. Int. J. Antimicrob. Agents.

[43] Jeong T.H., Hong H.W., Kim M.S., Song M., Myung H. (2023). Characterization of Three Different Endolysins Effective against Gram-Negative Bacteria. Viruses.

[44] Park D.W., Park J.H. (2020). Characterization of Endolysin LysECP26 Derived from rV5-like Phage vB_EcoM-ECP26 for Inactivation of Escherichia coli O157:H7. J. Microbiol. Biotechnol..

[45] Gerstmans H., Duyvejonck L., Vazquez R., Staes I., Borloo J., Abdelkader K., Leroy J., Cremelie E., Gutierrez D., Tames-Caunedo H. (2023). Distinct mode of action of a highly stable, engineered phage lysin killing Gram-negative bacteria. Microbiol. Spectr..

